# Fermentation-Derived Albumin-Based Hydrogels for Tissue Adhesion Applications

**DOI:** 10.3390/polym15112530

**Published:** 2023-05-31

**Authors:** Francesca Della Sala, Birgitte Mølholm Malle, Luigi Ambrosio, Assunta Borzacchiello

**Affiliations:** 1 Institute of Polymers, Composites and Biomaterials, National Research Council (IPCB-CNR), Viale J.F. Kennedy 54, 80125 Naples, Italy; francesca.dellasala@cnr.it (F.D.S.); luigi.ambrosio@cnr.it (L.A.); 2 Ascendis Pharma A/S, Tuborg Boulevard 12, 2900 Hellerup, Denmark; bmm@ascendispharma.com

**Keywords:** albumin, tissue adhesive, mechanical properties

## Abstract

Currently, most of the clinically available surgical glues and sealants lack elasticity, good adhesion and biocompatibility properties. Hydrogels as tissue adhesives have received extensive attention for their tissue-mimicking features. Here, a novel surgical glue hydrogel based on a fermentation-derived human albumin (rAlb) and biocompatible crosslinker for tissue-sealant applications has been developed. In order to reduce the risks of viral transmission diseases and an immune response, Animal-Free Recombinant Human Albumin from the saccharomyces yeast strain was used. A more biocompatible crosslinking agent, 1-ethyl-3-(3-dimethylaminopropyl) carbodiimide (EDC), was used and compared with glutaraldehyde (GA). The design of crosslinked albumin-based adhesive gels was optimized by varying the albumin concentration, the mass ratio between albumin and the crosslinking agent as well as the crosslinker type. Tissue sealants were characterized in terms of mechanical (tensile and shear), adhesive and in vitro biocompatibility properties. The results indicated that the mechanical and adhesive properties improved as the albumin concentration increased and the mass ratio between albumin and crosslinker decreased. Moreover, the EDC-crosslinked albumin gels have better biocompatibility properties than GA-crosslinked glues.

## 1. Introduction

Over the past three decades, advancements in medical technology have affected the development of modern surgical techniques. Nevertheless, wound leakage remains a common complication in general surgery. Indeed, the classic approaches of tissue sealing, such as sutures, staples or wires, although feasible in general surgery, cannot be applied in a minimally invasive way. Moreover, they can cause further tissue damage, increasing the risk of infection and, in some cases, fail to provide complete and instant tissue sealing. Various bioadhesive surgical glues have been used as a support or as an alternative to conventional methods of tissue sealing. [1]. Surgical glues are a class of biomaterials able to adhere to the tissues and are bioadhesive materials which polymerize rapidly, sealing the tissues or wounds in an atraumatic manner [2,3,4]. Tissue sealants have been used to promote tissue adhesion in hemostasis and the sealing of body fluids in surgery, wound closure, implant fixation, wound healing and fistula repair in a variety of surgical procedures [5]. Overall, the benefits of using surgical glues are their greater ease of application compared with traditional non-invasive sealing techniques, controllable mechanical strength and elasticity. They may also play an active role in the tissue-healing mechanism [6,7]. Ideally, surgical glue should be easy to apply and possess the following qualities: strong binding strength with the ability to form a watertight seal, mechanical compliance with the matrix, elasticity and biocompatible and biodegradable qualities which ultimately increase patient compliance with a minimal inflammatory reaction [8]. Surgical glues are based on the principles of bioadhesion, which is the ability to adhere to biological materials and to be retained on the biological substrate for a specific period [9]. The mechanism of adhesion is based on both chemical and physiological interactions. Chemical interactions involve the formation of ionic or chemical bonds between the interfaces of adhesives and biological substrate due to the interaction of different functional groups present in adhesives. The main three steps involved in chemical interactions are the wetting and swelling of the polymer and the interdiffusion between the polymer chain and the mucosal membrane followed by the formation of chemical bonds between the entangled chains. On the other hand, physiological interactions involve adhesion through physiologically related mechanisms such as the blood clotting process [2]. On the basis of these interactions and the type of biomaterial for the production of surgical glues, sealants can be formed by using natural or synthetic polymers, or a combination of both, such as fibrin, cyanoacrylates, polyethylene glycol-based adhesives and albumin. However, most tissue adhesives existing in the market do not offer both elasticity and good adhesion, and further problems of toxicity have been revealed. For example, cyanoacrylates, such as the cyanoacrylate adhesive Dermabond, have been successfully used to seal skin due to their high stiffness and adhesion strength, but they possess low elasticity and, furthermore, their degradation products, such as formaldehyde, are toxic to the human body. In contrast, fibrin-based sealants, mainly composed of virus-inactivated fibrinogen and serine protease factor XIII, are among the most successful adhesives so far due to their excellent biocompatibility, biodegradation and hemostasis, possessing more flexibility like the soft tissue. However they show low stiffness and weak adhesion strength, retaining the issue of the potential transmission of viral diseases [10]. Thus, novel types of tissue adhesives with good biocompatibility still need to be developed. Hydrogels as tissue adhesives in biomedical applications have received extensive attention due to their extracellular-matrix-mimicking characteristics [11]. Among the natural-based surgical glues, albumin is a globular protein found in blood plasma and is obtained from different animal sources. Various commercialized albumin glues (e.g., BioGlue^®^) have been FDA-approved and are used as hemostats in vascular and cardiac surgeries [12,13,14]. These products are generally composed of glutaraldehyde (GA) and bovine albumin available in a dual-chambered syringe. Animal-derived albumin sealants are easy to use, resistant and affordable, but they present several risks regarding the transmission of viral diseases and the toxicity of degradation products, as well as safety concerns over the use of crosslinkers such as GA [15]. Indeed, aldehyde-containing adhesives inevitably cause tissue necrosis and inflammation in the short-term as well as carcinogenicity in the long-term [16]. To overcome these drawbacks, the aim of this work was to design novel fermentation-derived human-albumin-based hydrogels with a biocompatible crosslinkers for tissue-sealant applications. Animal-Free Recombinant Human Albumin from the saccharomyces yeast strain (hereon named rAlb or Alb) has been produced with recombinant DNA techniques in order to reduce the risks of transmission of viral pathologies and immune response. In this work, rAlb was crosslinked with either GA as a control or a more biocompatible crosslinking agent, 1-ethyl-3-(3-dimethylaminopropyl) carbodiimide (EDC). The design of the crosslinked (CL) rAlb-based adhesive gels was optimized by varying the rAlb concentration, the rAlb and crosslinking agent mass ratio (crosslinking degree) and the crosslinker type. Tissue-adhesive systems can play a major role in biomedical applications if they possess adequate mechanical behavior and stability over time [17,18,19,20]. To this aim, the mechanical properties of the CL–rAlb gels and their dependence on the rAlb concentration, the crosslinking degree and the crosslinker type, were investigated by tensile and rheological tests. The optimization of the novel surgical glue properties was also performed in terms of the adhesion capability of the gels on different substrates (biological and non-biological). Lastly, in order to assess their potential effectiveness in the field of biomedical applications, the biological response of the CL–rAlb gels was verified by cytotoxicity tests.

## 2. Materials and Methods

### 2.1. Materials

rAlb was kindly provided by Novozymes Biopharma (Frederiksberg, Denmark). Glutaraldehyde (GA) and 1-Ethyl-(3-dimethylaminopropyl) carbodiimide (EDC), obtained from Sigma-Aldrich (St. Louis, Missouri, USA), were used as crosslinkers (CL). Phosphate buffered saline (PBS) tablets without calcium and magnesium were obtained from MP Biomedicals Inc. (Santa Ana, CA, USA).

### 2.2. Albumin Sample Preparation

The samples were prepared using a starting rAlb solution obtained by mixing 2.5 g of rAlb and 3.8 mL of water and magnetically stirring the solution for 20 h at room temperature (RT). The rAlb concentration in the starting solution was 336 mg/mL; this solution was diluted in order to obtain different rAlb concentrations. Afterward, the crosslinker and water were mixed and added to the starting rAlb solution in order to obtain the CL–rAlb gel. The CL–rAlb gels were prepared using different concentrations of rAlb and different rAlb/CL mass ratios (m.r.) as reported in Table 1.

**Figure 1:**
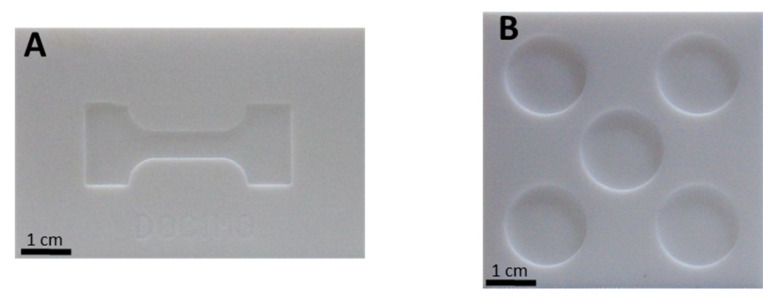
Mold characterized by a dog-bone shape for the Tensile Test (**A**) and mold with disc shape for the Rheological Test (**B**); scale bar = 1 cm.

To prepare samples for tensile tests to evaluate the mechanical properties of the gels, the starting rAlb solution, crosslinker and water were mixed and loaded into a dog-bone-shaped mold to obtain specimens with a rectangular section of dimensions 20 mm × 5 mm and a thickness of 1 mm. (Figure 1A). After crosslinking for 2 h, the samples were stored in PBS in order to prevent the gel from drying out. For rheological measurements, the starting rAlb solution, crosslinker and water were first mixed and then placed in a mold with a disc shape (Figure 1B) characterized by a diameter of 15 mm and a depth of 1 mm. The samples were left for 2 h at RT for complete crosslinking and then placed in PBS.

### 2.3. Characterization of Mechanical Properties

#### 2.3.1. Tensile Test

The mechanical properties of the CL–rAlb gels were evaluated by means of tensile tests performed using an Instron Machine (mod 5566) loaded with a cell of 100 N. The samples were placed between the grips of the dynamometer, and they were pulled at a speed of 1 mm/min. This method was adapted from the ASTM International Standard D882-02 “Standard Test Method for Tensile Properties of Thin Plastic Sheeting”. From these tests, stress–strain curves were obtained, and stress–strain values at break of CL–rAlb gels as a function of rAlb concentration and rAlb/CL mass ratio were evaluated. The tests were repeated three times for each sample.

#### 2.3.2. Rheological Test

Viscoelastic properties were evaluated by rheological tests performed on a rotational rheometer (MARS rheometer ™ III, HAAKE ™). The rheological tests were performed on gel discs with a diameter of 10 mm and a thickness of 1 mm. First, an amplitude sweep test was conducted at 1 Hz frequency to identify the range of the linear viscoelasticity regime. Afterward, the rheological tests were performed at a controlled temperature of 37 °C by using a thermostatic bath and using a plate–plate geometry (15 mm diameter) with a fixed amplitude in the 0.1–10 Hz frequency range. Oscillatory shear deformation in this frequency range was chosen since these can emulate the actual mechanical stresses occurring during normal physiological processes. To avoid water evaporation during the tests, the samples were placed in a special humidity chamber, and the relative humidity was set at 90%. The CL–rAlb gels were subjected to periodic oscillation in a dynamic experiment (small-amplitude frequency sweep tests) to evaluate the dependence of the elastic and viscous moduli, G′ and G″, on the frequency. G′ gives information about the elasticity or the energy stored in the material during deformation, whereas G” describes the viscous character or the energy dissipated as heat. In particular, the elastic modulus gives information about the capability of the sample to sustain a load and return to its initial configuration after an imposed stress or deformation [21].

### 2.4. Characterization of Adhesive Properties

**Figure 2:**
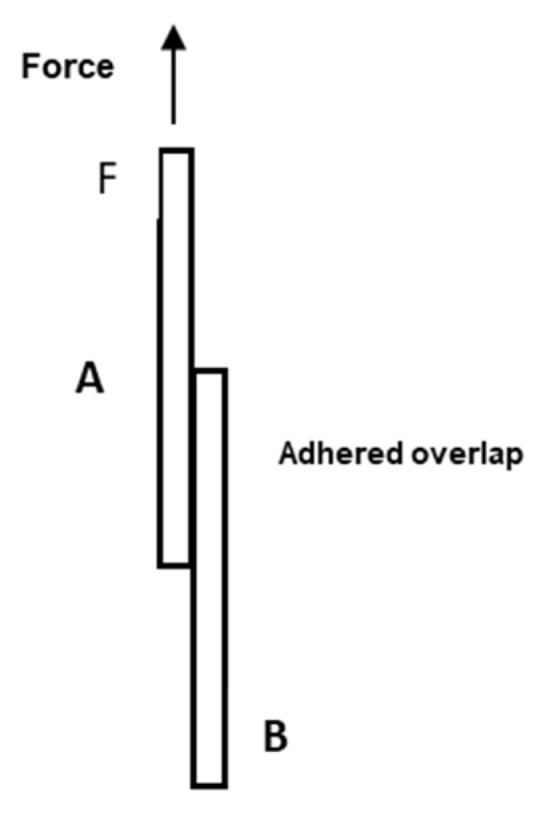
Schematic representation of the sample preparation.

In order to study the adhesion strength of CL–rAlb gels on biological (pig skin) and non-biological (high density polyethylene, HDPE) substrates, rectangular-shaped substrates of size 20 mm × 10mm × 1 mm were used. Pig skin was obtained from the slaughterhouse and used within 3 h. Solutions of rAlb and crosslinker were prepared and then quickly mixed; a volume of 20 μL of the mixed rAlb/CL solution was placed on an area of 100 mm^2^ of the substrate A, and it was subsequently covered with a second identical substrate B (Figure 2).

The bonding strength of adhesive–substrate samples was measured by means of an Instron Machine (mod. 5566) using a load cell of 100 N. In particular, the free surfaces of the substrates (A and B) were placed between the grips of the Instron machine and the shear force was applied by pulling the substrate A at an extension rate of 1 mm/min (Figure 2). This method was adapted from the ASTM International Standard D2339-98 “Standard Test Method for Strength Properties of Adhesives in Two-Ply Wood Construction in Shear by Tension Loading”. The separation force, obtained by the dynamometer, was divided by the contact area to yield the shear strength Σ. The tests were repeated at least three times on each sample.

### 2.5. Biological Properties

#### 2.5.1. Cell Culture

To test the biological properties of rAlb samples, primary human dermal fibroblasts (HDF, provided by Lonza) were used. HDF cells were cultured to passage 6–7 in a complete medium, composed of Eagle’s minimal essential medium (EMEM) supplemented with 20% Fetal Bovine Serum (FBS), 100 U/mL penicillin, 100 U/mL streptomycin and 2× non-essential amino acids. HDF cells were maintained in 100 mm diameter cell culture dishes in a humidified and controlled atmosphere at 37 °C and 5% CO_2_. The medium was changed every 3–4 days.

#### 2.5.2. Cell Viability

To investigate the cells’ viability, the gels were exposed to the cell culture medium for 42 h. The eluate thus obtained was incubated with HDF cells for the indirect cytotoxicity tests performed at 1 and 3 days. HDF cells were seeded at a density of 8 × 10^4^ cells/mL on 96-well plates (World Precision Instruments, Inc.). The cells were seeded in each well in triplicate and cultured for up to 72 h, and then an Alamar blue assay (AB) was performed by adding AB reagent to the samples (at 10% *v*/*v* with respect to the medium) and incubated at 37 °C for 4 h, in accordance with the ISO 10993–5: 2009 [22]. The absorbance of the samples was measured using a spectrophotometer plate reader (Multilabel Counter, 1420 Victor, Perkin Elmer) at 570 nm and 600 nm. AB is an indicator dye that incorporates an oxidation–reduction indicator that changes color in response to the chemical reduction in the growth medium resulting from cell viability. HDF-seeded wells exposed to fresh cell culture medium not previously incubated with the gels were used as a control. Data are expressed as the percentage difference between the treated and control cells to evaluate the percentage of reduction (Reduction %), which is calculated with the following formula:

$$Reduction\;(\%)=\frac{\left(O_{2}\times A_{1}\right)-(O_{1}\times A_{2})}{\left(O_{2}\times P_{1}\right)-(O_{1}\times P_{2})}\times 100$$

where *O*_1_ is the molar extinction coefficient (*E*) of oxidized AB at 570 nm; *O*_2_ is the *E* of oxidized AB at 600 nm; *A*_1_ is the absorbance of test wells at 570 nm; *A*_2_ is the absorbance of test wells at 600 nm; *P*_1_ is the absorbance of control wells at 570 nm; and *P*_2_ is the absorbance of control wells at 600 nm. The percentage reduction for each sample was normalized to the percentage reduction of the control at each time point to obtain the percentage cell viability for each sample [23].

### 2.6. Statistical Analyses

All the experiments were performed in at least three parallel groups, and the results in the figures are shown as mean ± SD. Data analysis was performed using Graphpad software. The repeated results were compared with the ordinary one-way analysis of variance ANOVA (Tukey’s multiple comparison test), and a *p*-value < 0.05 was considered significant.

## 3. Results and Discussion

### 3.1. Mechanical Properties: Tensile and Rheological Analysis

**Figure 3:**
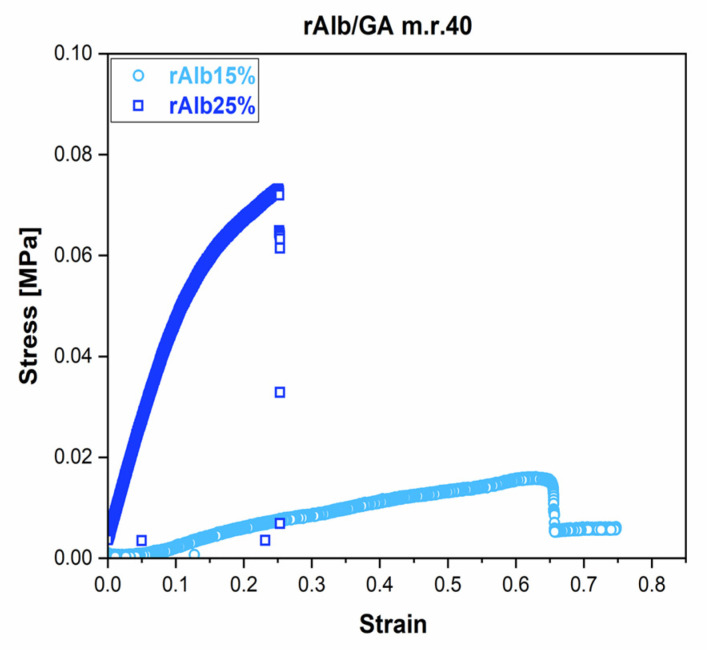
Representative stress versus strain curve for rAlb-gels with an rAlb concentration of 15% (round symbol) or 25% (square symbol) and an rAlb/GA mass ratio of 40.

A representative stress–strain curve obtained by tensile tests of CL–rAlb gels with an rAlb concentration of 15% or 25% and an rAlb/GA m.r. of 40 is shown in Figure 3. Typically, the stress–strain curve shows three regions: The first is a non-linear region in which the material shows a high deformation when applying a low stress [24]. This behavior could be explained by considering a possible rearrangement of polymeric chains within the gel. The second part of the curve is characterized by an elastic response of the material, showing a linear relationship between the applied stress and the deformation of the material, while the third part of the curve is a plastic region until a maximum stress value is reached. After this value, there is a sudden reduction in stress, meaning that there was breakage of the sample. The maximum stress values, σ max, for all samples is reported in Table 2.

For both types of crosslinkers and for different alb/CL mass ratios, increasing the rAlb concentration increases the maximum stress value. In particular, when EDC is used as the crosslinker, for samples having an Alb/CL m.r. of 10, increasing the rAlb concentration from 15% to 25% increases the σmax from 4 × 10^−2^ ± 1 × 10^−2^ to 6 × 10^−2^ ± 2 × 10^−2^ MPa, respectively, while for samples having an Alb/CL m.r. of 5, the σmax increases from 1 × 10^−1^ ± 1 × 10^−2^ to 3 × 10^−1^ ± 2 × 10^−2^ MPa. Similarly, when using GA as the crosslinker, the increase in rAlb concentration from 15% to 25% results in an increase in σmax from 2 × 10^−2^ ± 1 × 10−^2^ to 7 × 10^−2^ ± 1 × 10^−2^ MPa, respectively, when the Alb/CL m.r. is 40, and from 1 × 10^−1^ ± 2 × 10^−3^ to 2 × 10^−1^ ± 3 × 10^−2^ MPa when the Alb/CL m.r. is 20. The increase in rAlb/CL mass ratio implies a decrease in the maximum stress value for both types of crosslinkers and for both rAlb concentrations. In particular, considering EDC as the crosslinker, increasing the rAlb/CL m.r. from 5 to 10 decreases the maximum σmax stress from 4 × 10^−2^ ± 1 × 10^−2^ to 1 × 10^−1^ ± 1 × 10^−2^ MPa for the samples at an rAlb concentration of 15% and from 6 × 10^−2^ ± 2 × 10^−2^ to 3 × 10^−1^ ± 2 × 10^−2^ MPa when the rAlb concentration is 25%. When GA is used as crosslinker, the σmax decreases from 2 × 10^−2^ ± 1 × 10^−2^ MPa (alb/CL m.r. of 40) to 1 × 10^−1^ ± 2 × 10^−3^ MPa (alb/CL m.r. of 20) for the samples at an rAlb concentration of 15%, and from 7 × 10^−2^ ± 1 × 10^−2^ (alb/CL m.r. of 40) to 2 × 10^−1^ ± 3 × 10^−2^ MPa (alb/CL m.r. of 20) for the samples at an rAlb concentration of 25%. Furthermore, the rAlb-based gels prepared using GA reach lower maximum stress values and lower deformation at break in comparison with the samples prepared with a high mass ratio of EDC.

**Figure 4:**
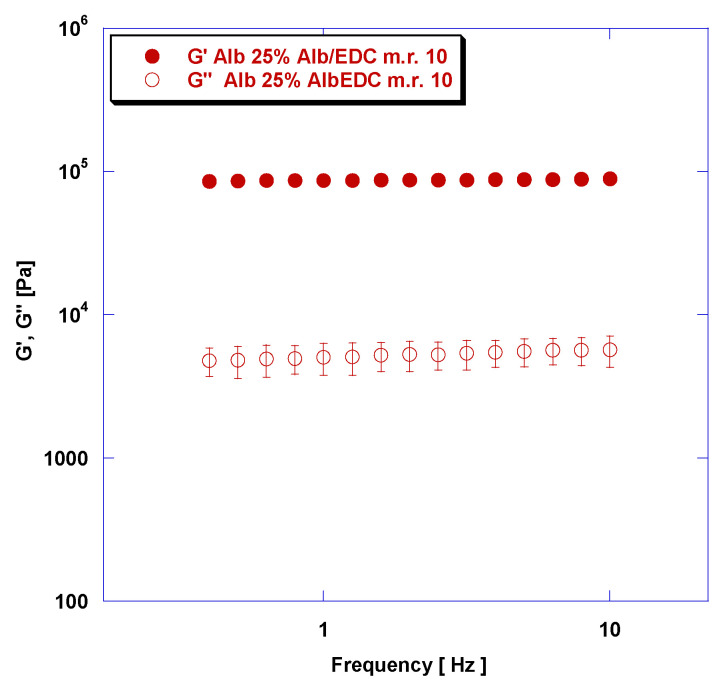
The elastic and viscous moduli versus frequency for a sample at an rAlb concentration of 25% and an rAlb/EDC mass ratio of 10. The sample was 15 mm in size with a thickness of 1 mm. Data represented as mean ± SD (*n* = 3).

A representative mechanical spectrum, which shows the dependence of G′ and G″ as a function of frequency for rAlb-based gels, is reported in Figure 4.

In particular, the mechanical spectra related to the samples is characterized by an rAlb concentration of 25% and an rAlb/EDC mass ratio of 10. It can be noted that G′ > G″ (G′ is higher than G″ by at least one order of magnitude) and that G′ and G″ are almost independent from frequency. This data suggest that the viscoelastic properties of rAlb-based gels show a “strong gel” behavior. The rheological behavior of these materials is mainly attributable to the existence of covalent-type chemical bonds between the rAlb molecules due to the presence of the crosslinking agent. The presence of these chemical bonds causes a reduction in the intrinsic motility of the polymer chains which does not allow the material to relax the applied stresses. Consequently, the network polymer reacts to the applied stresses mainly by deforming elastically.

**Figure 5:**
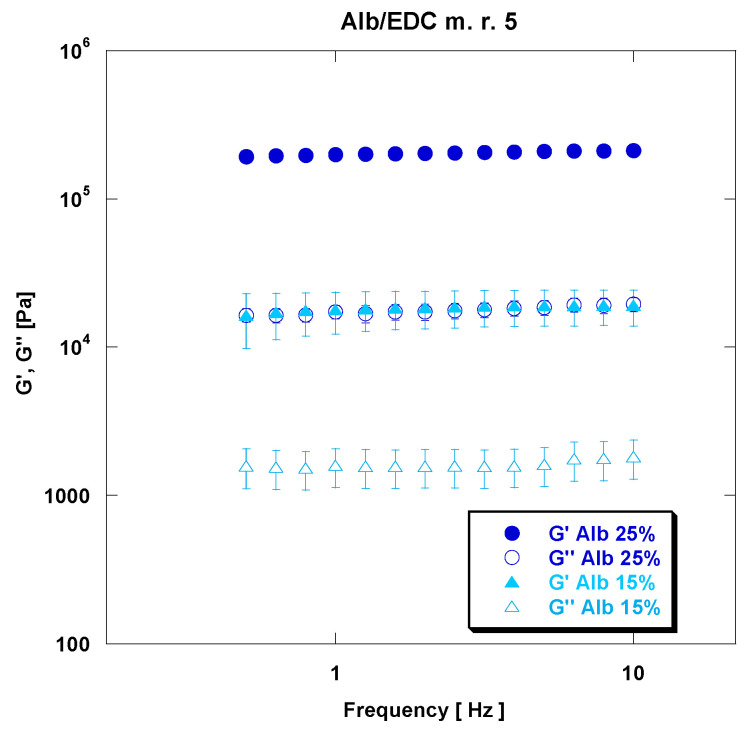
Representative mechanical spectra of rAlb-based gels for samples at an rAlb concentration of 15% and 25% and at an rAlb/EDC mass ratio of 5. The sample was 15 mm in size with a thickness of 1 mm. Data represented as mean ± SD (*n* = 3).

Figure 5 shows the comparison between representative gels at an rAlb concentration of 15% and 25% and an rAlb/EDC mass ratio of 5, and the effect of rAlb concentration on the viscoelastic properties of the rAlb-based gels is reported.

From this figure, it can be noticed that increasing the rAlb concentration, while keeping the rAlb/CL mass ratio constant, indicates an increase in viscoelastic properties. In particular, at an rAlb/EDC mass ratio of 5, the values of G′ and G″ at a frequency of 5 Hz are 1.85 × 10^4^ MPa and 1.58 × 10^3^ MPa for the sample at an rAlb concentration of 15%, and 2.09 × 10^5^ MPa and 1.83 × 10^4^ MPa for the sample at an rAlb concentration of 25%. Thus, increasing the rAlb concentration from 15% to 25% increases the value of G′ by one order of magnitude. We can conclude that, as reported in Table 3, increasing the rAlb concentration positively influences the viscoelastic properties; indeed, an increase in rAlb concentration leads to an increase in viscoelastic parameters by one order of magnitude.

**Figure 6:**
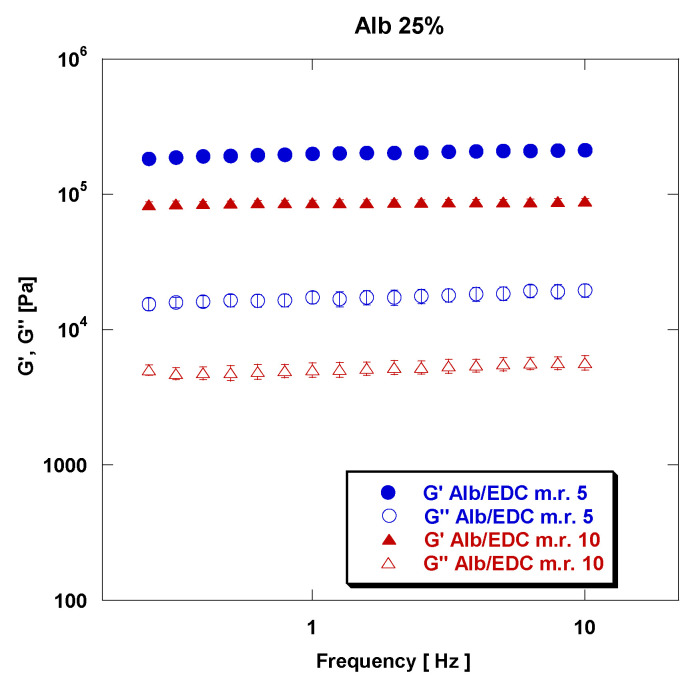
Effect of the rAlb/CL mass ratio on rheological properties of rAlb-based gels for samples at an rAlb/EDC mass ratio of 5 and 10 and at an rAlb concentration of 25%. The sample was 15 mm in size with a thickness of 1 mm. Data represented as mean ± SD (*n* = 3).

The effect of the rAlb/CL mass ratio on the rheological properties of rAlb-based gels is reported in the representative Figure 6.

The elastic and viscous moduli of samples at an rAlb concentration of 25% and an rAlb/EDC mass ratio of 5 and 10 are reported in Figure 6. From this representative figure, it can be observed that decreasing the rAlb/CL mass ratio while keeping the rAlb concentration constant leads to an increase in the viscoelastic properties of the gels. In particular, as reported in Table 3, at an rAlb concentration of 15%, the values of G′ and G″ at a frequency of 5 Hz are 2.44 × 10^4^ MPa and 2.13 × 10^3^ MPa for the sample with an rAlb/GA mass ratio of 20, and 1.89 × 10^5^ MPa and 1.34 × 10^4^ MPa for the sample with an rAlb/GA mass ratio of 40. Thus, decreasing the rAlb/GA mass ratio from 40 to 20 increases the value of G′ by one order of magnitude.

**Figure 7:**
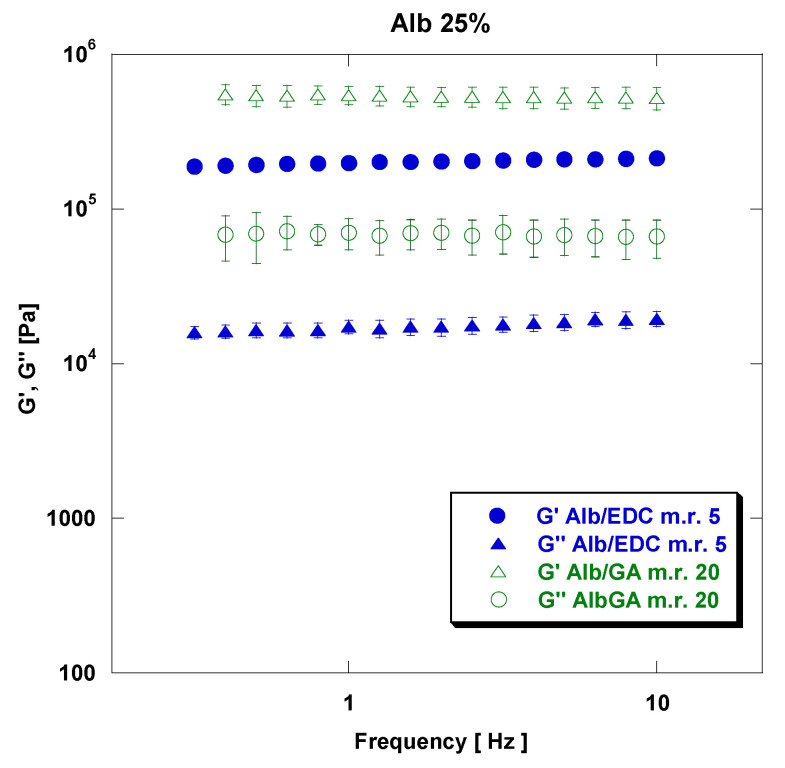
Effect of crosslinker type on rheological properties of rAlb-based gels for samples at an rAlb concentration of 25%, an rAlb/EDC mass ratio of 5 and an rAlb/GA mass ratio of 20. The sample was 15 mm in size with a thickness of 1 mm. Data represented as mean ± SD (*n* = 3).

The effect of the crosslinker type on the viscoelastic properties of rAlb-based gels is shown in Figure 7.

Figure 7 reports the comparison between the mechanical spectra obtained from samples crosslinked with EDC at an rAlb/EDC mass ratio of 5 (rAlb/EDC mole ratio of 0.015) and samples crosslinked with GA at an rAlb/GA mass ratio of 20 (rAlb/GA mole ratio of 0.030), at an rAlb concentration of 25%.

It is necessary to note that we have compared gels with the same concentrations of rAlb—15% and 25%, respectively—and with a molar ratio (m.r.) between rAlb and GA double that between rAlb and EDC, because GA binds reactive groups of Alb with double covalent bonds, while the EDC creates simple covalent bonds. From these figures, it can be noticed that gels crosslinked with GA show overall values of viscoelastic properties of the same order of magnitude as those prepared using EDC as the crosslinking agent (Table 3). In particular, for rAlb at 25% concentration, G′ and G″ values at a frequency of 5 Hz were 2.09 × 10^5^ MPa and 1.83 × 10^4^ MPa, respectively, for the sample with an rAlb/EDC mass ratio of 5 (rAlb/EDC mole ratio of 0.015), while G′ and G″ values for the sample with an rAlb/GA mass ratio of 20 (rAlb/GA mole ratio of 0.030) were 5.22 × 10^5^ MPa and 6.84 × 10^4^ MPa, respectively. These data suggest that rAlb-based gels crosslinked with GA show values of viscoelastic properties slightly higher than those prepared using EDC as a crosslinking agent.

The novel surgical glue produced shows a crosslinking process similar to that of commercial glue and is suitable for adhesive applications (data not shown) [25]. Mechanical characterization results demonstrate that, regardless of the crosslinking agent used, and with the same mass ratio between rAlb and CL, the increase in rAlb concentration from 15% to 25% corresponds to a significant increase in their elastic and viscous moduli, which is attributable to a greater number of bonds formed in the polymer network, and therefore to a greater stiffness of the material. Furthermore, this effect is more evident for higher values of the mass ratio between rAlb and CL, and for gels obtained using GA as a crosslinking agent. For both rAlb concentrations, 15% and 25%, the gels made using GA as the crosslinking agent show comparable elastic and viscous moduli to those obtained using EDC. Furthermore, the mechanical spectra trends differ more significantly for high rAlb concentrations and for low mass ratios between rAlb and CL. Understanding the rheology of a tissue adhesive can provide insight into its cohesive response when under mechanical stress, which is important in understanding its clinical effectiveness [24,26,27]. Dynamic mechanical analysis can be described by the shear storage modulus G′ and the shear loss modulus G″. These parameters provide information on adhesion capability and should ideally be balanced so as not to create an adhesive that is either too elastic or too brittle, which may result in suboptimal adhesive strength. This indicates that, in general, a tissue adhesive’s mechanical strength may rely upon an “optimum range” of G′ and G″, which may not necessarily be the highest value of G′ and G″, in line with previous research on tissue adhesive rheology [28]. Indeed, for example, a polymer solution will not generate sufficient adhesion, and, similarly, in stiffer gels, polymer chains are entrapped in the network and lack the mobility to interact with the biological substrate and to promote adhesion [29,30]. Comparing the results obtained here from the tensile and rheological tests, it can be noted that, for all rAlb concentrations and for the different mass ratios between rAlb and the crosslinking agent, the gels made using GA as a crosslinking agent show comparable or slightly higher values of elastic and viscous moduli but lower tensile strength values than those obtained for gels made using EDC. Therefore, GA-crosslinked rAlb gels are able to withstand lower tensile loads.

### 3.2. Characterization of Adhesive Properties

**Figure 8:**
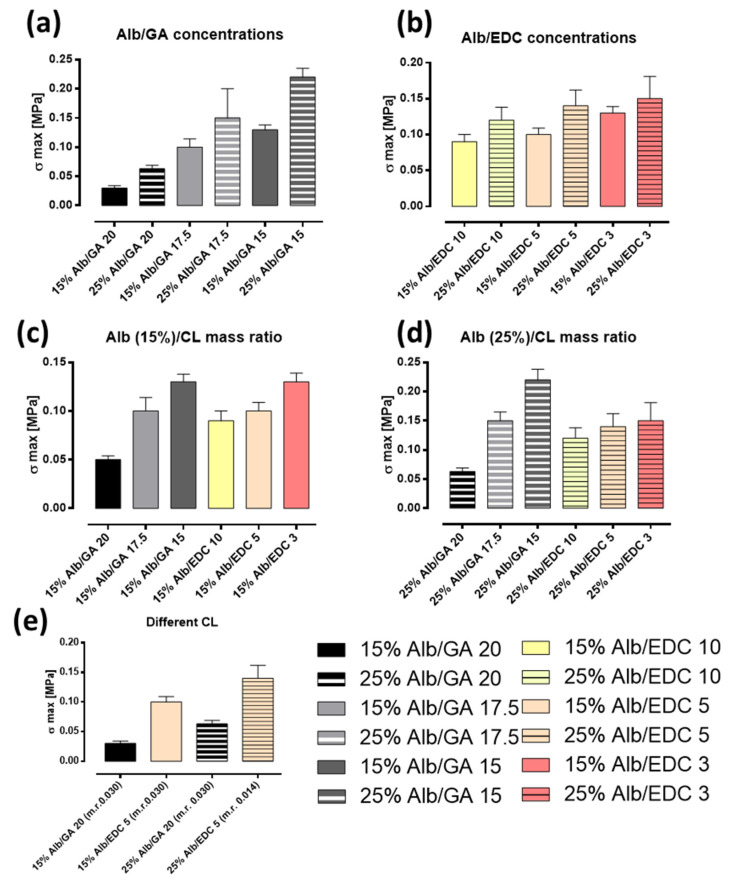
Adhesive properties on HDPE. (**a**) Effect on σ max of rAlb concentration/GA. (**b**) Effect on σ max of rAlb concentration/EDC. (**c**) Effect on σ max of the mass ratio between rAlb and CL (15% rAlb). (**d**) Effect on σ max of the mass ratio between rAlb and CL (25% rAlb). (**e**) Comparison between the σ max obtained for the different CLs. The sample size was 20 mm × 5 mm with a thickness of 1 mm. Data represented as mean ± SD (*n* = 3).

To characterize the adhesive properties of the gels prepared, tests were carried out on two different substrates: HDPE and pig skin. With the aim of quantitatively analyzing the adhesive bond, the σmax, obtained from the stress–strain curves relative to the shear tests carried out on each sample, was used as an indicative parameter of adhesive strength. In Figure 8a,b, the effect of rAlb concentration on the adhesion process of the gels is shown, and the maximum stress values, σ max, are reported. As can be seen from the histograms, the adhesive strength increases with increasing an rAlb concentration from 15% to 25% for both crosslinking agents, GA (Figure 8a) and EDC (Figure 8b). The effect of the rAlb/CL mass ratio on the adhesion process of the gels is displayed in Figure 8c,d; specifically, the maximum stress values, σ max, are reported. As can be seen from the figures, on the HDPE substrate, the adhesive strength of the gels increases with increasing mass ratio Alb/CL for both 15% (Figure 8c) and 25% (Figure 8d) rAlb concentrations for both crosslinking agents (GA and EDC) used. Finally, Figure 8e shows a comparison between the two different crosslinkers. The σmax of gels with equal concentrations of rAlb and a molar ratio (m.r.) between rAlb and GA double that of rAlb and EDC were compared. It was evident that, for both rAlb concentrations, the samples made using EDC had superior adhesive properties compared with those obtained using GA.

**Figure 9:**
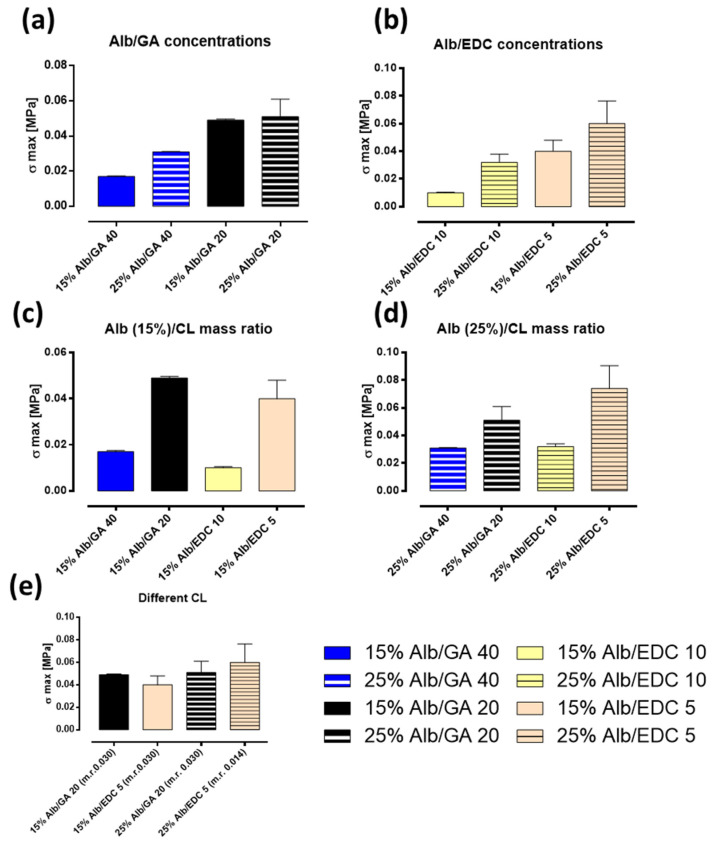
Adhesive properties on pig skins. (**a**) Effect on σ max of rAlb concentration/GA. (**b**) Effect on σ max of rAlb concentration/EDC. (**c**) Effect on σ max of the mass ratio between rAlb and CL (15%rAlb). (**d**) Effect on σ max of the mass ratio between rAlb and CL (25% rAlb). (**e**) Comparison between the σ max obtained for the different CLs. The sample size was 20 mm × 5 mm with a thickness of 1 mm. Data represented as mean ± SD (*n* = 3).

Similarly to the adhesion tests carried out on HDPE, adhesion tests on pig skin were analyzed by estimating the maximum stress that the specimen is able to withstand when subjected to shear, as this represents an indicative parameter of the adhesion strength between the adhesive and the support. The following histograms (Figure 9) show the shear strength values for all the gels made using either GA or EDC as the crosslinking agent. These confirm what has already been observed from the results obtained for HDPE; specifically, that the adhesive strength is greater when the rAlb concentration is higher. Once again, we wanted to investigate the dependence of σmax on the mass ratio between rAlb and CL. To this end, the values of the maximum stresses obtained for the samples containing the same rAlb concentrations were compared. These comparisons were made for rAlb concentrations of 15% (Figure 9c) and 25% (Figure 9d) and for different mass ratios between rAlb and CL. At both rAlb concentrations, and for both crosslinking agents used, as the mass ratio between rAlb and CL decreases, or as the concentration of CL increases, the resistance of the adhesive to shear stress improves. Moreover, similarly to the results obtained using HDPE, on pig skin, the samples made using EDC had similar or superior adhesive properties compared with those obtained using GA. The testing of adhesive properties showed that tissue adhesives-based on an rAlb have very promising properties for bioadhesive applications, combining viscoelasticity properties useful for dissipating energy upon adhesive debonding and much stronger interactions with the substrates [31,32]. An increase in the rAlb concentration has a positive effect on the adhesion properties of the gel as higher values of σmax are obtained. In addition, decreasing the rAlb/CL mass ratio increases the amount of CL, and this positively influences the adhesion properties of the gel because σmax increases.

### 3.3. Biological Properties

**Figure 10:**
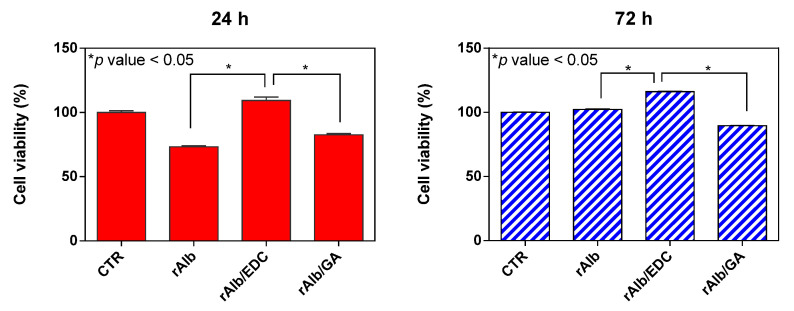
Alamar Blu assay at 24 h (red) and 72 h (blue) of HDF cell culture seeded on gels. The sample size was 20 × 5 mm with a thickness of 1 mm. Data represented as mean ± SD (*n* = 3).

In order to investigate the biocompatibility of the gels, HDF cell viability was evaluated by the Alamar Blue assay. The metabolic activity of the cells was measured at 24 and 72 h of cell culture. The irreversible reaction of resazurin to resorufin, which takes place during this test, provides an indirect indication of the cell viability over the time points. As can be observed from Figure 10, the gels showed good biocompatibility after 24 h and 72 h of cell culture compared with the control. Indeed, gels showed a cell viability percentage of approximately 70% for rAlb alone, 100% for rAlb/EDC and 80% for rAlb/GA at 24 h of culture. After 72 h, the cell percentage viability was higher compared with the 24 h time point: all samples showed a cell viability percentage of approximately 100% due to the increase in proliferation of the cells. In any case, the cell viability percentage after 24 h and 72 h was higher in the sample with rAlb and EDC as the crosslinker compared with rAlb alone or rAlb with GA as the crosslinker. Biocompatibility is an important criterion for the applicability of a tissue sealant. This is more relevant for the CL sealant as degradation products of this crosslinking agent have been shown to produce foreign body reactions [33]. GA is a widely used crosslinking agent for improving the mechanical properties and resistance to enzymatic degradation of biomaterial devices, but several drawbacks such as calcification and cytotoxicity have been reported [34]. It is well known that EDC modifies side groups on proteins, making them reactive with other side groups and mediating the ester bond formation between the hydroxyl and carboxyl groups. In contrast to conventional chemical agents such as GA, EDC does not remain as a part of that linkage but simply changes to water-soluble urea derivatives. Thus, the use of EDC should not present cytotoxic effects, and these residues can be metabolized by the cell [33]. These results are in accordance with the literature and demonstrate that the use of EDC as a crosslinker is preferable to maintain an optimal level of biocompatibility of the cells compared with the use of GA as a crosslinking agent [35,36].

## 4. Conclusions

In this work, the mechanical characterization, in terms of both tensile and rheological properties, and adhesive properties together with the biocompatibility test of rAlb-based gels were analyzed in order to optimize their characteristics and potential in bioadhesive applications. The tissue adhesives were obtained by adding the crosslinking agents EDC and GA to rAlb solutions. Different types of tissue adhesive samples were produced by suitably varying chemical–physical parameters such as the concentration of rAlb, the degree of crosslinking and the type of crosslinking agent. From the mechanical characterization results, it can be concluded that, regardless of the type of crosslinking agent used, the mechanical properties improve as the rAlb concentration increases and the mass ratio between rAlb and the crosslinking agent decreases. Moreover, it also emerged that EDC-crosslinked rAlb gels have enhanced mechanical properties than those obtained using GA. From the rheological analysis carried out, it was noted that the tissue-adhesive gels behaved like “strong gels”. In particular, the samples made using GA as the crosslinking agent showed comparable or slightly higher elastic and viscous moduli than those obtained using EDC. The overall mechanical behavior of the EDC-crosslinked samples is better than that of GA-crosslinked gels. Finally, the adhesive properties of the gels on HDPE substrates and pig skin were investigated. This analysis confirmed the results obtained from the mechanical tests, indicating that the maximum adhesion strength is higher when using EDC as the crosslinker on both biological and non-biological substrates. Lastly, the biocompatibility test showed a higher percentage of cell viability of EDC-crosslinked rAlb compared with the samples containing GA as a crosslinker. In conclusion, in view of future applications in the biomedical field, it will be necessary to evaluate these tissue adhesives in in vivo models to better understand their effective biocompatibility and, specifically, the hemostasis and wound-healing capabilities of these adhesives to allow tissue regeneration after surgeries. While this needs to be explored in the near future, these results prove that the optimization of parameters including rAlb concentration, mass ratio and crosslinker types is essential for designing more performant rAlb-based tissue adhesives.

## Figures and Tables

**Table 1:** Description of samples.

Albumin Concentration (*w*/*v*)	Crosslinker (CL)	Albumin/CL Mass Ratio (Molar Ratio)
15%	EDC	10 (0.030)
		5 (0.015)
		3 (0.010)
	GA	40 (0.060)
		20 (0.030)
		15 (0.020)
25%	EDC	10 (0.030)
		5 (0.015)
		3 (0.010)
	GA	40 (0.060)
		20 (0.030)
		15 (0.020)

**Table 2:** Maximum stress values for all samples.

Albumin Concentration (*w*/*v*%)	Crosslinker	Albumin/CL Mass Ratio	σmax [MPa]
15%	EDC	10	4 × 10^−2^ ± 1 × 10^−2^
25%	EDC	10	6 × 10^−2^ ± 2 × 10^−2^
15%	EDC	5	1 × 10^−1^ ± 1 × 10^−2^
25%	EDC	5	3 × 10^−1^ ± 2 × 10^−2^
15%	GA	40	2 × 10^−2^ ± 1 × 10^−2^
25%	GA	40	7 × 10^−2^ ± 1 × 10^−2^
15%	GA	20	1 × 10^−1^ ± 2 × 10^−3^
25%	GA	20	2 × 10^−1^ ± 3 × 10^−2^

**Table 3:** rAlb-based sample description and related viscoelastic properties at a frequency of 5 Hz.

Albumin Concentration (*w*/*v*%)	Crosslinker	rAlb/CL Mass Ratio	G′, Pa at 5 Hz	G″, Pa at 5 Hz
15%	EDC	10	6.06 × 10^3^	1.27 × 10^3^
25%	EDC	10	8.71 × 10^4^	5.36 × 10^3^
15%	EDC	5	1.85 × 10^4^	1.58 × 10^3^
25%	EDC	5	2.09 × 10^5^	1.83 × 10^4^
15%	GA	40	7.22 × 10^3^	3.29 × 10^2^
25%	GA	40	1.89 × 10^5^	1.35 × 10^4^
15%	GA	20	2.44 × 10^4^	2.13 × 10^3^
25%	GA	20	5.22 × 10^5^	6.84 × 10^4^

## Data Availability

Not applicable.
